# Diffuse Unilateral Subacute Neuroretinitis: Challenges in Diagnosis and Management

**DOI:** 10.7759/cureus.58510

**Published:** 2024-04-18

**Authors:** Mustafa Nurul-Farhana, Abdul Aziz Roslin-Azni, Tan Sor-Earn, Ismail Shatriah, Oli Mohamed Shelina

**Affiliations:** 1 Department of Ophthalmology and Visual Sciences, School of Medical Sciences, Universiti Sains Malaysia, Kubang Kerian, MYS; 2 Department of Ophthalmology, Hospital Shah Alam, Shah Alam, MYS; 3 Ophthalmology, Eyemedics Specialist Eye Centre, Kota Kinabalu, MYS; 4 Department of Ophthalmology, Faculty of Medicine, Universiti Teknologi MARA, Sungai Buloh, MYS

**Keywords:** exudative retinal detachment, laser prp, migrating choroiditis, worm, diffuse unilateral subacute neuroretinitis

## Abstract

We report two cases of diffuse unilateral subacute neuroretinitis (DUSN) where multimodal imaging was used to assist in locating the nematode. The first case presented with clusters of migrating choroiditis with a suspicious tiny visualized worm noted on serial fundus photography. The second case had an atypical presentation with extensive exudative retinal detachment and a suspicious coiled worm in the subretinal space noted on optical coherence tomography. Both cases received oral albendazole for six weeks while the first case received additional argon laser photocoagulation to the suspected nematode. Both cases showed resolution of the ocular inflammation upon completion of treatment with no further recurrences. DUSN should be suspected in young and healthy patients with unexplained unilateral inflammatory eye disease with severe loss of vision. This series highlights the challenges faced in identifying the nematode in cases with suspected DUSN.

## Introduction

Diffuse unilateral subacute neuroretinitis (DUSN) is an ocular parasitic infection that can result in severe visual loss characterized by multifocal chorioretinitis caused by a variant of nematode species [1-4].^ ^DUSN usually occurs in healthy children and young adults in two clinical stages.

Diagnosing DUSN is often challenging with smaller-sized nematodes when it is difficult to visualize the worm. To date, there is also no standard protocol for the treatment of DUSN. Whenever a nematode is visualized, focal laser photocoagulation is recommended for the area of the worm [5]. We present two cases of DUSN with initial challenges in the diagnosis and identification of the nematode. Visualization of the suspected nematode was assisted using multimodal imaging in both cases. The first case illustrates a late presentation resulting in poor visual gains following laser photocoagulation and medical treatment. The second case highlights a missed initial diagnosis due to an atypical presentation resulting in irreversible visual loss despite medical treatment.

## Case presentation

Case 1

**Figure 1:**
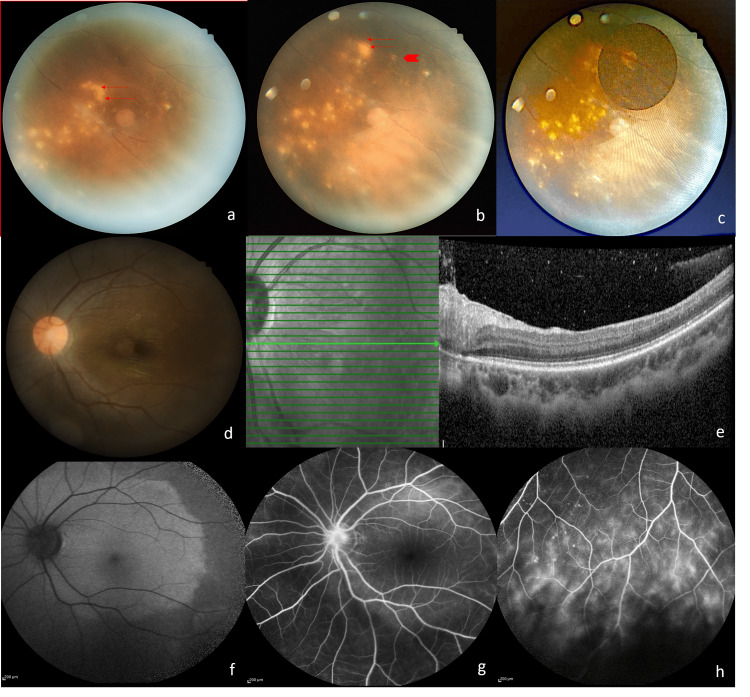
(a) Clusters of grayish-yellow subretinal lesions inferotemporally with vascular sheathing and vitritis with the subretinal track (double arrows). (b) Change in the location and pattern of the subretinal track (double arrows). Coiled worm around a retinal vessel (thick arrow). (c) Enlarged view of the worm. (d) Hyperemic optic disc with an epiretinal membrane. (e) OCT showing hyperreflective dots in the vitreous and epiretinal membrane. (f) FAF showing increased autofluorescence signal at the peripapillary and macula region. (g) FFA showing a hot disc. (h) FFA showing inferior vasculitis with staining of the outer retinal lesions. OCT = optical coherence tomography; FAF = fundus autofluorescence; FFA = fundus fluorescein angiography

**Figure 2:**
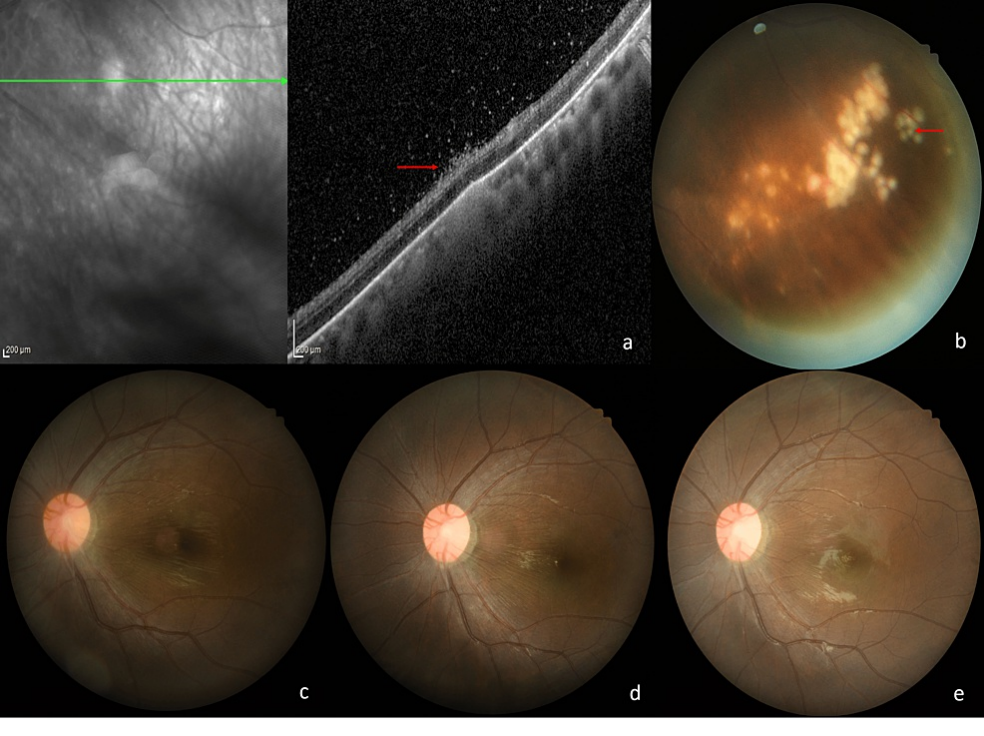
(a) OCT at the inferotemporal retina showing retinal thinning with focal hyperreflectivity above the RPE (triangular arrow), increased hyperreflectivity over the inner retinal surface (red arrow), and hyperreflective dots in the vitreous. (b) Laser photocoagulation over the nematode (arrow) and the clusters of adjacent subretinal lesions. (c) Fundus image at presentation showing a hyperemic optic disc with epiretinal membrane and normal vessel caliber. (d, e) Optic disc pallor 6 and 18 months later with attenuation of arterioles seen nasally. OCT = optical coherence tomography; RPE = retinal pigment epithelium

A healthy nine-year-old boy presented with left eye redness for one month associated with progressive blurring of vision for three days. His symptoms began six months before presentation with intermittent floaters and blurring of vision. On presentation, visual acuity in the left eye was 1/60 and 6/9 in the right eye. There was a grade 1 relative afferent pupillary defect (RAPD) in the left eye. Examination of the left eye revealed 3+ anterior chamber cells and 2+ anterior vitreous cells. Posterior segment examination showed vitritis grade 1, hyperemic optic disc with an epiretinal membrane over the macula (Figure 1, Panel d), and clusters of grayish-white subretinal lesions inferiorly with vascular sheathing (Figure 1, Panel a). Serial fundus examination showed a changing pattern and location of the suspicious subretinal track (Figure 1, Panel b), adjacent new clusters of subretinal lesions at the inferotemporal region, surrounding pigmentary retinal pigment epithelial degenerative changes, and a suspicious small C-shaped white lesion resembling a coiled worm seen near the track (Figure 1, Panels b and c). The suspicious worm was immobile with no photosensitivity reaction noted. Optical coherence tomography (OCT) of the macula showed hyperreflective dots in the vitreous and an epiretinal membrane (Figure 1, Panel e). OCT at the inferotemporal retina showed generalized retinal thinning with hyperreflectivity over the inner retinal surface and more hyperreflective dots in the vitreous (Figure 2, Panel a). Fundus fluorescein angiography (FFA) of the left eye showed a hot disc (Figure 1f), and vasculitis predominantly inferiorly with staining of the subretinal lesions (Figure 1, Panel g).

Blood parameters were normal with no eosinophilia and negative infective screening. The toxoplasma serology was negative. He was treated with oral albendazole 200 mg twice daily for six weeks and an immunosuppressive dose of oral corticosteroids tapered over six weeks. Topical dexamethasone was prescribed for the left eye. Laser photocoagulation was given directly to the entire worm and the clusters of adjacent subretinal lesions in a single setting (Figure 2, Panel b). A spot size of 200 μm with power between 150 and 200 mW and a duration of exposure of 0.2 seconds was used. The entire worm was covered with confluent white laser marks. On follow-up, the left visual acuity showed improvement but remained at 6/45 secondary to the optic nerve dysfunction and presence of an epiretinal membrane. Fundus autofluorescence (FAF) at three weeks showed generalized increased autofluorescence at the peripapillary and macula region (Figure 1, Panel h). Inflammation of the left eye resolved with no further recurrences over six months. Optic disc pallor and some arteriolar attenuation were noted at six months which was more apparent at 18 months follow-up (Figure 2, Panels d and e).

Case 2

**Figure 3:**
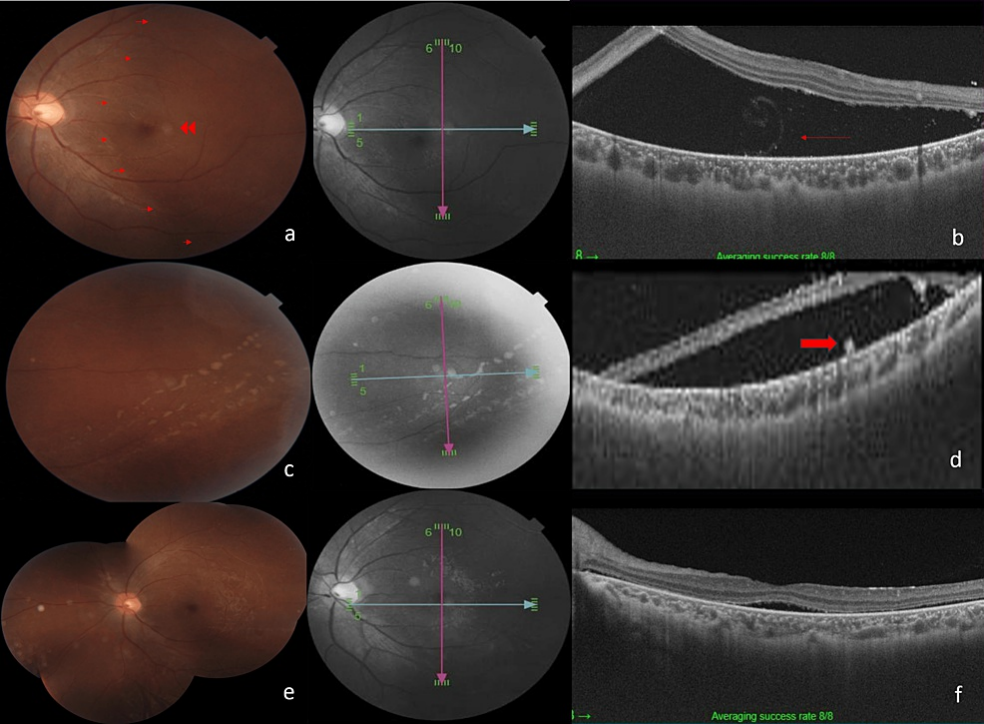
(a) Left exudative retinal detachment involving the macula (red triangular arrows), a suspicious white subretinal round lesion (double arrows). (b) OCT with gross subretinal fluid and confirmed coiled worm (red arrow) within. (c) Long subretinal track. (d) Serous detachment at the area of the subretinal track. Focal hyperreflectivity at the outer retina (arrow). (e) Fundus image six weeks post-treatment showing a hyperemic optic disc and vascular sheathing. (f) OCT showing resolving serous detachment. OCT = optical coherence tomography

A healthy 16-year-old boy with no underlying medical illness presented with a painless left eye with diminishing peripheral vision for one month associated with floaters. There was no eye redness, discharge, flashes of light, central vision loss, or reduced night vision and no preceding fever. He denied a history of ocular trauma or surgery. There was no family history of night blindness and no significant drug history. He frequently went with his father for camping trips. Visual acuity on presentation was 6/6 in the right eye and 6/7.5 in the left. The anterior chamber was deep and quiet. Intraocular pressures were normal. The lens was clear. There were anterior vitreous cells of 1+. Funduscopy showed a mildly hyperemic optic disc and vasculitis. There was no obvious retinitis, choroiditis, or retinal hemorrhages. The retina was otherwise flat. OCT of the macula was normal with no cystoid macula edema or subretinal fluid. Preliminary uveitic workup showed a normal full blood count except for borderline eosinophilia of 0.31, erythrocyte sedimentation rate of 14 mm/hour, negative tuberculosis Quantiferon, human immunodeficiency virus, and Venereal Disease Research Laboratory screening. An initial diagnosis of non-infectious intermediate uveitis was made. An anti-inflammatory dose of oral corticosteroids was prescribed. On review two weeks later, the left visual acuity had deteriorated to hand movements with a positive left RAPD. There were anterior chamber cells of 1+, vitritis 1+, and extensive exudative retinal detachment from 2 to 5 o’clock involving the macula (Figure 3a). There were no retinal breaks. Inferotemporally there was a long suspicious subretinal track with serous detachment (Figure 3c). No photosensitivity reaction or movement of the worms was noticed during a slit-lamp examination. OCT of the macula showed gross subretinal fluid with a suspicious coiled worm within (Figure 3b). OCT over the subretinal track showed gross subretinal fluid with focal hyperreflectivity (Figure 3d). The diagnosis was revised to DUSN. An urgent referral to the vitreoretinal surgeon was made. However, as the vision had rapidly deteriorated to no perception of light, no surgical intervention was planned. It was not possible to laser the nematode due to gross retinal detachment. He was managed conservatively with oral albendazole 400 mg twice daily and tapering oral corticosteroids for six weeks. He did not regain visual acuity but the detachment eventually resolved (Figures 3e, 3f).

## Discussion

DUSN is a clinical syndrome caused by a wandering worm in the subretinal space that induces inflammation of the ocular structures. The pathogenesis of DUSN seems to involve a local toxic effect on the retina from the worm by-products. Severe visual loss is attributed to progressive ganglion cell loss from the host’s immune reaction to the toxin [1,6,7]. Early presentation is characterized by diffuse intraocular inflammation with unexplained vitritis, papillitis, and migrating crops of multiple white evanescent retinal lesions. The presence of a subretinal track is suggestive of the movement of the nematode with local inflammation. In the later stages, severe visual loss, optic atrophy, narrowing of retinal vessels, and degeneration of retinal pigment epithelium are noted [8-13].

In this series, we have illustrated two cases of DUSN from Malaysia where the late presentation, delay in diagnosis, and difficulty in the identification of the nematode resulted in poor visual outcomes. In Case 1, the child presented six months after the onset of symptoms. The clusters of multifocal migrating yellowish lesions at the outer retina seen inferiorly with overlying vitritis were suggestive of DUSN. The use of serial fundus photography and OCT enabled better visualization of the nematode which was in the vicinity of the subretinal lesions and a subretinal track. The entire worm, which was lasered once identified, was not mobile and appeared to be of a smaller size measuring 400-700 µm, hence the difficulty in locating it. The exact subtype of the nematode was not known. The nematodes in DUSN can either be smaller (400-1,000 µm) or larger (1,500-2,000 µm) [4,13,14].

A diagnosis of presumed DUSN is made when the nematode is not located. It is based on characteristic clinical findings, as mentioned earlier. The diagnosis would be further supported by eosinophilia, positive serology for nematode, or visualization of the worm on an OCT scan with negative infective screening.

In our series, Case 2 had atypical clinical findings and the diagnosis of DUSN was initially missed. The diagnosis was only established later using multimodal imaging with serial fundus photos and OCT scans. By then, the patient had developed rapid, progressive, irreversible visual loss. The irreversible visual loss was attributed to the progression of extensive exudative retinal detachment involving the macula due to the migration of the nematode in the subretinal space. OCT of the macula showed a suspicious coiled nematode within the subretinal fluid. A long subretinal track was also seen on the fundus photos. This was supported by borderline high eosinophilia and negative infective screening. The size of the nematode seemed to be of the larger subtype.

The gold standard treatment for DUSN is laser photocoagulation of the worm. However, the worm is visualized in only 30% of cases [5,15]. Oral anti-helminthic agents are the mainstay of treatment in cases of presumed DUSN. Most reported cases used oral albendazole 400 mg daily for 30 days, and one study by Amaral et al. used only six days of oral albendazole 400 mg daily [16]. All cases showed good visual outcomes with no side effects. The efficacy is reported to be higher when there is a breakdown in the blood-retinal barrier, i.e., in eyes that have vitritis, as seen in Case 1. Laser photocoagulation performed in the vicinity of the subretinal lesions is also postulated to alter the blood-retinal barrier, thus improving ocular penetration of albendazole [11,15,17]. There is still no standard regimen for dosing and duration of oral albendazole for the treatment of DUSN. Other agents that have been used include thiabendazole and diethylcarbamazine [4,17].

In addition to albendazole, both cases concomitantly received a tapering anti-inflammatory dose of corticosteroids for six weeks. Resolution of ocular inflammation was seen. There is no standard protocol regarding the utilization of systemic corticosteroids in the treatment of DUSN. However, local inflammation from the by-products of the worm and toxic immune response known as toxic autoimmune nematode retinopathy may warrant the use of systemic corticosteroids to reduce the inflammation and improve the visual outcome [12,17,18]. Most reported cases that received oral corticosteroids showed greater visual improvement. A combination of laser photocoagulation, oral albendazole, and systemic steroids showed greater improvement in final visual acuity compared to each treatment in isolation.

The importance of oral albendazole in combination with oral corticosteroids in the treatment of DUSN when the worm was not lasered was illustrated in Case 2. Anatomical improvement was seen with the dosage and duration used but as expected there was no functional improvement. There were no side effects from treatment with albendazole such as stomach pain or nausea.

DUSN is also described as unilateral wipe-out syndrome as the disease eventually results in optic atrophy, narrowing of major vessels, and retinal pigment epithelial cell degeneration, leading to poor visual outcomes [16]. The degree of inflammation in DUSN depends on the immune response of the patient [18]. Intense inflammation from by-products will also lead to the death of ganglion cells, resulting in poor visual outcomes. Older age of more than 20 years and early presentation within one month have been associated with greater improvement in final visual acuity [17]. Other rare complications documented in the literature include choroidal neovascular membrane, disciform scar, and submacular granuloma [2,19].

The poor visual acuity gain following the commencement of treatment in Case 1 was explained by the delayed presentation, baseline papillitis, epiretinal membrane, and increased autofluorescence at the peripapillary and macula region noted on FAF indicating retinal pigment epithelial degeneration. During follow-up at six months, optic atrophy and attenuation of arterioles were noted. Table 1 describes a comparison of our cases with other published DUSN cases.

**Table 1:** Comparison of clinical presentation and treatment of DUSN with other reported cases. DUSN = diffuse unilateral subacute neuroretinitis; NPL = no light perception

Authors/Year	Age (years)	Duration of symptoms	Visual acuity (presentation)	Clinical presentation	Laser photocoagulation	Anti-helminth use	Steroid usage	Visual acuity (final)
Kang et al. 2015 [7]	52	10 days	6/60	Yellow-white infiltrative subretinal lesion superior to the fovea and worm inferotemporal to fovea	Yes	Albendazole 400 mg BD for 2 weeks	Yes	6/30
Curragh et al. 2018 [12]	8	2 months	6/12	Vitritis and migrating chorioretinitis with visualized worm	Yes	Albendazole 200 mg OD for 30 days	Yes	6/9
Ramachandran et al. 2020 [14]	45	4 days	6/9	A gray-white retinal lesion with subretinal tracks and visualized worm	Yes	Albendazole for 400 mg OD for 1 month	Yes	6/6
Amaral et al. 2021 [16]	51	3 weeks	CF	Vitritis, optic disc edema with macula star exudate, and migrating foci of retinitis	No	Albendazole 400 mg OD for 6 days	Yes	6/12
Mazzeo et al. 2021 [19]	16	4 months	6/30	Vitritis, hyperemic optic disc, submacular granuloma, and no worm visualized	No	Albendazole 400 mg OD for 30 days	No	6/24
Case 1	9	6 months	1/60	Vitritis and migrating peripheral choroiditis with visualized worm	Yes	Albendazole 200 mg BD for 6 weeks	Yes	6/45
Case 2	16	1 month	6/7.5	Vitiritis, extensive exudative retinal detachment, tracking retinal signs, and no worm visualized clinically	No	Albendazole 400 mg BD for 6 weeks	Yes	NPL

## Conclusions

DUSN often poses a diagnostic and therapeutic challenge. Late presentation, delay in diagnosis, and difficulty in identification of the worm are some of the challenges faced. The use of multimodal imaging may facilitate visualization of the nematode. Awareness of the variable clinical presentations of DUSN would enable an early diagnosis. A combination of treatment with laser photocoagulation to the identified worm together with high doses of albendazole and corticosteroids hasten visual recovery and yield better visual prognosis.
